# Pulmonary Embolism in Hematologic Malignancies: Predictive Value of the D-Dimer/Albumin Ratio and Proposal of the Hema-PE Score

**DOI:** 10.3390/jcm14207337

**Published:** 2025-10-17

**Authors:** Esma Sevil Akkurt, Ozlem Duvenci Birben, Mehmet Hakan Akbulut, Beyza Nur Ozturk, Aleyna Ozad, Mehmet Sinan Dal, Derya Yenibertiz

**Affiliations:** 1 Department of Pulmonology, Dr. Abdurrahman Yurtaslan Ankara Oncology Training and Research Hospital, 06200 Ankara, Türkiye; drozlemd.birben@gmail.com (O.D.B.); bbbeyza98@gmail.com (B.N.O.); aleynaozad18@gmail.com (A.O.); yenibertizderya@gmail.com (D.Y.); 2 Department of Hematology, Dr. Abdurrahman Yurtaslan Ankara Oncology Training and Research Hospital, 06200 Ankara, Türkiye; mhakanakbulut@gmail.com (M.H.A.); dr.sinandal@gmail.com (M.S.D.)

**Keywords:** hematologic malignancy, pulmonary embolism, risk assessment, D-dimer/albumin ratio

## Abstract

**Background:** Pulmonary embolism (PE) represents a major complication in patients with hematologic malignancies, yet existing risk assessment models such as the Khorana and ThroLy scores show limited applicability in this population. Novel tools incorporating routinely available clinical and laboratory markers are needed for accurate risk stratification. **Objectives:** To investigate the incidence and predictors of PE in patients with hematologic malignancies and to develop a new risk stratification model, the Hema-PE Score. **Methods:** This retrospective study included a total of 177 patients with various hematologic malignancies who were evaluated for, of whom 63 had pulmonary embolism (PE) and 114 served as controls. Clinical variables (immobility, central venous catheter) and laboratory markers (D-dimer/albumin ratio, hemoglobin, platelet count, CRP) were analyzed. Receiver operating characteristic (ROC) curve analyses were performed to assess predictive accuracy. A novel scoring system, the Hema-PE Score, was constructed and its performance compared with existing risk models. **Results:** PE was identified in 35% of patients. The D-dimer/albumin ratio showed strong discriminatory power for predicting PE (AUC = 0.82). Based on multivariable predictors, the Hema-PE Score was developed (range 0–7 points). At a threshold of ≥3, the score achieved 100% sensitivity and 76% specificity (AUC = 0.88). Compared with the Khorana and ThroLy scores, the Hema-PE Score demonstrated superior predictive performance across hematologic malignancy subtypes. **Conclusions:** The D-dimer/albumin ratio and the newly developed Hema-PE Score demonstrated strong predictive performance for pulmonary embolism in patients with hematologic malignancies. These findings suggest that the Hema-PE Score may serve as a practical and easily applicable risk stratification tool, supporting early diagnosis and guiding thromboprophylaxis decisions in clinical practice. Prospective multicenter validation studies are warranted to confirm its utility and to facilitate its integration into patient management strategies.

## 1. Introduction

Venous thromboembolism (VTE), including deep vein thrombosis (DVT) and pulmonary embolism (PE), is a major cause of morbidity and mortality in patients with malignancy. While the incidence of VTE is well established in solid tumors, patients with hematological malignancies are also at significant risk [1,2]. Several factors, such as advanced age, immobility, central venous catheters, infections, and chemotherapy regimens, have been associated with increased thrombotic risk in this population [3]. Importantly, patients with hematological malignancies present unique challenges due to concomitant cytopenias and a higher bleeding risk, which complicates thromboprophylaxis strategies.

In recent years, the incidence of cancer-associated thrombosis in patients with hematologic malignancies has been increasingly recognized, with rates often exceeding those observed in solid tumors. Beyond disease-related factors, systemic inflammation, endothelial injury, and malnutrition play central roles in the development of a prothrombotic state. Elevated inflammatory cytokines and acute-phase reactants contribute to hypercoagulability, while low serum albumin reflects both nutritional and inflammatory burden. These interrelated mechanisms highlight the need for comprehensive biomarkers that capture multiple aspects of thrombogenesis. The D-dimer/albumin ratio represents such an integrative marker, reflecting the combined effects of coagulation activation and systemic inflammation. However, its clinical utility and predictive performance in hematologic malignancies remain poorly defined.

Risk assessment models such as the Khorana score, widely used in solid tumors, and the ThroLy score, designed for lymphoma patients, have provided valuable insights into VTE prediction [4,5]. However, their applicability across the heterogeneous group of hematological malignancies—including leukemia, lymphoma, and multiple myeloma—remains limited. This highlights the need for tailored tools that incorporate both clinical and laboratory parameters to better stratify thrombotic risk in this population [6]. Unlike traditional scores such as Khorana and ThroLy, which rely heavily on tumor type and platelet count, the D-dimer/albumin ratio simultaneously captures hypercoagulability, inflammation, and nutritional status—three major contributors to thrombosis in hematologic malignancies.

In this context, we developed the **Hema-PE Score (Hematological Malignancy–Pulmonary Embolism Scoring System)**, a novel hybrid model integrating clinical risk factors and a biomarker (D-dimer/albumin ratio). The present study aimed to evaluate the incidence of PE in patients with hematological malignancies, analyze associated risk factors, and validate the diagnostic performance of the Hema-PE Score.

## 2. Materials and Methods

### 2.1. Study Design and Population

This retrospective study included patients aged ≥18 years who were diagnosed and followed with hematological malignancies at the Hematology and Pulmonology clinics of Dr. Abdurrahman Yurtaslan Ankara Oncology Training and Research Hospital between 1 January 2019 and 31 December 2024. A total of 177 patients were enrolled, of whom 63 patients with confirmed PE constituted the case group, while 114 patients without PE served as the control group. Eligible diagnoses included leukemia, lymphoma, and multiple myeloma (MM). Acute myeloid leukemia (AML) and acute lymphoblastic leukemia (ALL) were categorized as acute leukemias, while Hodgkin and non-Hodgkin lymphomas were grouped under lymphomas. Patients with benign hematological disorders or those receiving anticoagulant therapy for other indications (e.g., cardiovascular disease or prior thrombosis) were excluded. Variables significantly associated with PE in univariate analysis were considered for score development. Details of the score construction are presented in Section 3. All consecutive adult patients diagnosed with hematologic malignancies who underwent thoracic CT imaging for suspected thromboembolic events or other clinical indications were screened for eligibility.

### 2.2. Data Collection

Clinical and laboratory data were obtained retrospectively from electronic hospital records (FONET system) and patient files. Collected variables included demographic features, comorbidities, smoking status, laboratory findings (complete blood count, coagulation parameters, and biochemical tests at admission), pathology reports, treatment regimens, and imaging results (venous Doppler ultrasonography and thoracic computed tomography). The presence and location of pulmonary embolism were confirmed by CT pulmonary angiography. Patients without radiological or clinical evidence of PE throughout follow-up served as the control group. D-dimer values were expressed in mg/L FEU and albumin values in g/dL. Immobility ≥ 3 days was defined according to nursing documentation or physician notes indicating bed rest or restricted mobility due to illness, treatment, or hospitalization.

### 2.3. Statistical Analysis

Data were analyzed using IBM SPSS Statistics version 25.0 (IBM Corp., Armonk, NY, USA). Descriptive statistics were presented as **n (%) for categorical variables** and as **mean ± standard deviation or median (min–max) for continuous variables**, as appropriate. The **Shapiro–Wilk test** was applied to assess the normality of continuous variables. For group comparisons, the **Pearson’s chi-square test** or **Fisher’s exact test** was used for categorical variables, and the **Mann–Whitney U test** was applied for non-normally distributed continuous variables. The predictive performance of the D-dimer/albumin ratio and the Hema-PE Score was evaluated using **receiver operating characteristic (ROC) curve analyses**, with calculation of sensitivity, specificity, positive predictive value (PPV), negative predictive value (NPV), and area under the curve (AUC). **Kaplan–Meier survival analysis** was planned for prognostic evaluation. All statistical analyses were conducted using **two-sided tests**, and a ***p*-value < 0.05** was considered statistically significant.

### 2.4. Ethics Statement

The study protocol was approved by the Ethics Committee of Dr. Abdurrahman Yurtaslan Ankara Oncology Training and Research Hospital on 06 March 2025 (Approval No: 2025-03/47). Written informed consent forms were distributed to and signed by all participants (or their legal guardians) prior to participation. The study was conducted in accordance with the principles of the Declaration of Helsinki. As this was a retrospective chart review, informed consent was waived.

## 3. Results

### 3.1. Patient Characteristics

A total of 177 patients were included in the study; 63 developed pulmonary embolism (PE) and 114 served as controls. The median age was 66 years in the PE group and 63 years in the control group. There were no significant differences between the groups regarding sex, smoking status, history of myocardial infarction or stroke, or mediastinal/extranodal lymph node involvement (*p* > 0.05). Among patients with PE, the most frequent hematologic malignancies were multiple myeloma (46.0%) and lymphoma (36.5%). PE was bilateral in 57.1% of cases, right-sided in 27.0%, and left-sided in 15.9%. Massive PE occurred in 7.9% of patients, submassive in 1.6%, and non-massive in 90.5%. Concomitant DVT was observed in 14.3% of PE cases. The presence of comorbidities was significantly higher in the PE group (*p* < 0.001) (Table 1).

### 3.2. D-Dimer/Albumin Ratio

**Figure 1:**
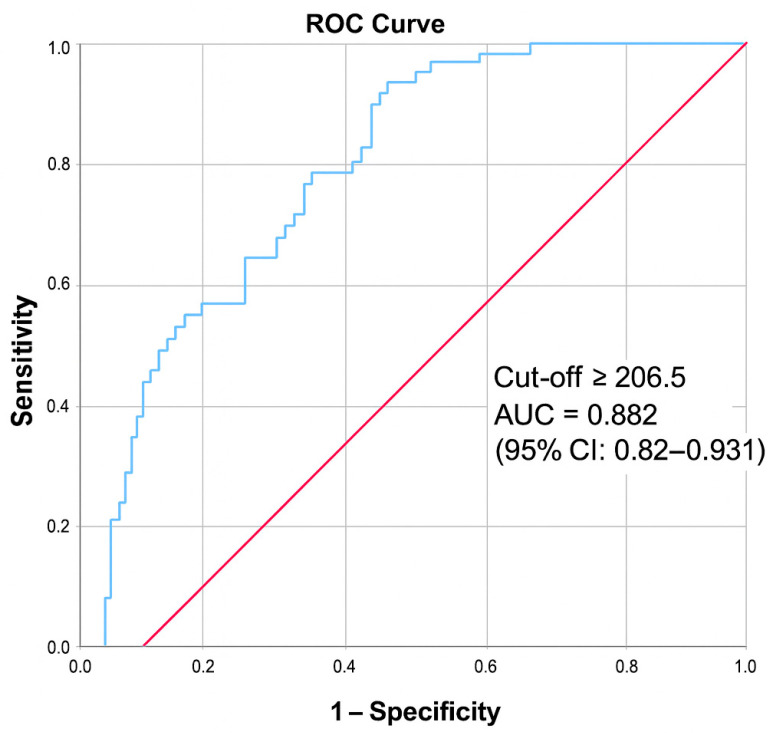
ROC curve of the D-dimer/Albumin ratio in predicting pulmonary embolism.

The median D-dimer/albumin ratio was significantly higher in patients with PE compared with controls (*p* < 0.001). ROC curve analysis demonstrated that a ratio ≥ 206.5 predicted PE with 100.0% sensitivity and 76.3% specificity. The area under the ROC curve (AUC) was 0.882 (95% CI: 0.832–0.931; *p* < 0.001), indicating good discriminative ability (Table 2, Figure 1).

### 3.3. Development of the Hema-PE Score

Based on univariate analyses, six parameters were identified as significant predictors of pulmonary embolism (PE) and were selected for the construction of the Hema-PE Score: D-dimer/albumin ratio, immobility, central venous catheter, C-reactive protein (CRP), platelet count, and hemoglobin (Table 3). To construct a simple and practical risk assessment tool, one point was assigned to each independent predictor, except for the D-dimer/albumin ratio, which was weighted with two points due to its higher odds ratio and stronger effect size compared with other predictors in the multivariate analysis. Cut-off values for continuous variables were determined using ROC curve analysis for the D-dimer/albumin ratio. Established thresholds from previous studies were used for hemoglobin (<10 g/dL), platelet count (>350 × 10^9^/L), and CRP (>10 mg/L).

Univariate analyses demonstrated that immobility ≥ 3 days, presence of a central venous catheter, low hemoglobin (<10 g/dL), elevated platelet count (>350 × 10^9^/L), elevated CRP (>10 mg/L), and an increased D-dimer/albumin ratio (>206.5) were significantly associated with pulmonary embolism (all *p* < 0.05). These variables were therefore selected for inclusion in the Hema-PE Score.

The final scoring system ranged from 0 to 7 points, stratifying patients into three categories: low risk (0–2 points), intermediate risk (3–4 points), and high risk (5–7 points). The distribution of the Hema-PE Score differed significantly between groups: the majority of PE patients were classified as intermediate-to-high risk (≥3 points), whereas most controls were in the low-risk category (≤2 points).

**Figure 2:**
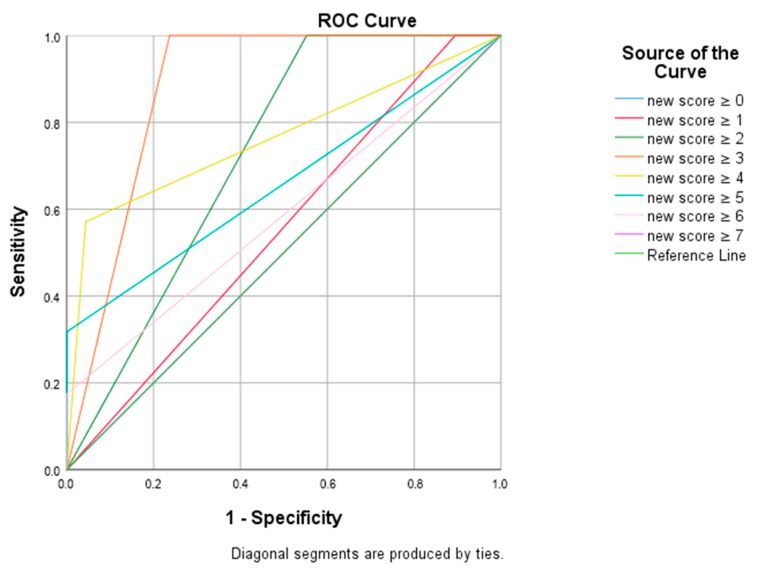
ROC curve of the new scoring system in predicting pulmonary embolism.

ROC analysis demonstrated that a cut-off score of ≥2 yielded a sensitivity of 100.0% and a specificity of 44.7% (AUC: 0.724, *p* < 0.001). A score of ≥3 provided the best balance between sensitivity and specificity, with an AUC of 0.882 (95% CI: 0.832–0.931; *p* < 0.001), 100.0% sensitivity, and 76.3% specificity. Higher cut-off values (≥4 and ≥5) further increased specificity (>95%) at the expense of sensitivity. The diagnostic performance of the scoring system at different cut-off points is presented in Table 4 and illustrated in Figure 2.

## 4. Discussion

In this study, we evaluated the incidence and predictors of pulmonary embolism (PE) in patients with hematologic malignancies and developed a novel risk stratification tool, the Hema-PE Score. PE occurred at a relatively high rate (35%) in this population, emphasizing its clinical significance. Both the D-dimer/albumin ratio and the Hema-PE Score demonstrated strong discriminatory ability, suggesting potential value for early diagnosis and risk assessment.

Previous studies have reported PE incidence rates of 5–15% in hematologic malignancies [7,8]. The higher rate in our cohort may reflect more advanced disease, frequent central venous catheter use, and pro-inflammatory conditions. This underscores that thrombotic risk in hematologic cancers varies considerably depending on disease type and clinical context.

D-dimer and albumin have been individually associated with cancer-related thrombosis [9,10,11]. D-dimer reflects hypercoagulability, while hypoalbuminemia indicates systemic inflammation and malnutrition. Our findings show that the D-dimer/albumin ratio is a strong predictor of PE in hematologic malignancies. The ratio reflects both hypercoagulability and systemic inflammation/nutritional status, integrating multiple thrombogenic mechanisms into a single, easily obtainable biomarker. Since both parameters are routinely measured, the ratio represents a simple and readily applicable tool for clinical practice.

The Hema-PE Score combines clinical and laboratory variables that represent key mechanisms of thrombus formation. D-dimer/albumin captures hypercoagulability and inflammation; immobility and central venous catheters are established clinical risk factors; and elevated CRP, anemia, and thrombocytosis reflect pro-thrombotic states in cancer. The hemoglobin and CRP thresholds were based on established literature (<10 g/dL and >10 mg/L, respectively) to ensure clinical applicability.

Compared with existing models, the Hema-PE Score offers several advantages. The Khorana Score, developed for solid tumors, and the ThroLy Score, specific to lymphoma, have limited predictive value across hematologic subtypes [1,5]. In contrast, the Hema-PE Score integrates both clinical and laboratory parameters, improving applicability across the full spectrum of hematologic malignancies.

The Hema-PE Score achieved 100% sensitivity at a ≥3 threshold, supporting its potential use as a screening tool. However, this finding should be interpreted cautiously, as it may reflect overfitting due to the small sample size and single-center design. Subgroup analyses by leukemia, lymphoma, and myeloma were attempted but underpowered. Higher thresholds (≥4 or ≥5) improved specificity (>95%), indicating flexibility for diverse clinical contexts and risk-benefit considerations.

All components of the Hema-PE Score are objective and routinely measured, enhancing reproducibility. However, reliance on static cut-offs (e.g., CRP, hemoglobin, platelet count) may reduce accuracy in patients with borderline values or confounding conditions such as infection or chemotherapy-induced cytopenias [12,13]. Future studies should explore whether dynamic, serial assessment of the score provides better predictive performance.

Beyond its predictive performance, the Hema-PE Score may also have implications for personalized clinical management in hematologic malignancies. Integrating such a score into clinical workflows could support early identification of high-risk patients, facilitate timely diagnostic imaging, and optimize allocation of prophylactic strategies. Moreover, incorporating laboratory-based indices with clinical data aligns with the growing trend toward precision medicine and may complement artificial intelligence–driven risk assessment models currently under development. Future research combining hematologic, inflammatory, and metabolic parameters could further refine this approach, leading to more individualized and cost-effective preventive strategies. In this context, the Hema-PE Score represents not only a prognostic indicator but also a potential framework for dynamic risk monitoring in routine hematology practice.

Clinically, the Hema-PE Score may assist in bedside decision-making when imaging is not immediately available and may help generate hypotheses regarding thromboprophylaxis, although it should not be interpreted as a direct therapeutic recommendation. Its strong sensitivity at lower thresholds supports its use for screening, while higher cut-offs can identify patients at very high risk who may benefit from closer monitoring.

Prediction models are essential not only to guide prophylaxis but also to balance thrombotic and bleeding risks. Anticoagulant-related bleeding substantially increases mortality in patients with hematologic malignancies and cancer-associated thrombosis, particularly intracranial and gastrointestinal bleeding [14,15]. Recent studies have further emphasized the need for refined risk assessment tools that incorporate inflammatory and nutritional markers in this population [6,10]. Individualized risk stratification is therefore critical to optimize benefit and minimize harm.

This study has several limitations. First, its retrospective, single-center design may limit generalizability. Second, although the sample size was sufficient for preliminary evaluation, subgroup analyses by malignancy subtype were not feasible. Third, heterogeneity in treatment regimens and clinical characteristics could have influenced thrombotic risk. The relatively high PE incidence may reflect selection bias due to inclusion of patients who underwent CT imaging. In addition, the model was not internally validated (e.g., by bootstrapping or cross-validation), and potential confounders such as chemotherapy, immunotherapy, and infection were not fully adjusted for. Finally, external validation was not performed. Future multicenter prospective studies are warranted to confirm the predictive value of the Hema-PE Score and support its incorporation into clinical practice.

In conclusion, our study identifies the D-dimer/albumin ratio as a promising biomarker and introduces the Hema-PE Score as a practical, effective tool for predicting PE in hematologic malignancies. If validated prospectively, it could complement current risk models, enable earlier diagnosis, and inform thromboprophylaxis strategies in this high-risk population.

## 5. Conclusions

The present study suggests that the D-dimer/albumin ratio may represent a useful biomarker, and that the newly developed Hema-PE Score shows promising predictive performance for pulmonary embolism in patients with hematologic malignancies. If validated in prospective multicenter cohorts, this simple and practical score could potentially complement existing risk assessment strategies, support earlier diagnosis, and contribute to guiding prophylactic interventions in clinical practice. Beyond its diagnostic potential, the Hema-PE Score may also enhance individualized patient care by integrating routine laboratory and clinical parameters into a single, accessible framework. Broader implementation of such models could ultimately improve early recognition, risk stratification, and preventive management of thromboembolic events in hematology practice.

## Figures and Tables

**Table 1:** Demographic and Clinical Characteristics of the Patients.

(**A**) Baseline demographics.			
**Variable**	**Control Group (n = 114)**	**Patient Group (n = 63)**	***p***
Age [years, median (min–max)]	62	66	0.530
Sex	Female: 44 (38.6%) Male: 70 (61.4%)	Female: 27 (42.9%) Male: 36 (57.1%)	0.580
Smoking (n, %)	44 (38.6%)	22 (34.9%)	0.628
History of MI or stroke (n, %)	9 (7.9%)	2 (3.2%)	0.332
(**B**) Disease-related features.			
**Variable**	**Control Group (n = 114)**	**Patient Group (n = 63)**	***p***
Disease type (n, %)	Leukemia: 29 (25.4%) Lymphoma: 28 (24.6%) MM: 57 (50.0%)	Leukemia: 11 (17.5%) Lymphoma: 23 (36.5%) MM: 29 (46.0%)	<0.001 *
Mediastinal LN involvement (n, %)	No: 8 (28.6%) Yes: 20 (71.4%)	No: 4 (20.0%) Yes: 16 (80.0%)	0.737
Extranodal involvement (n, %)	No: 8 (28.6%) Yes: 20 (71.4%)	No: 3 (35.0%) Yes: 17 (65.0%)	0.755
PE location (n, %)	-	Right: 17 (27.0%) Left: 10 (15.9%) Bilateral: 36 (57.1%)	-
PE severity (n, %)	-	Massive: 5 (7.9%) Submassive: 1 (1.6%) Nonmassive: 57 (90.5%)	-
DVT (n, %)	-	Present: 9 (14.3%)	-

* *p* < 0.001 indicates statistical significance.

**Table 2:** Cut-off values and ROC analysis results of the D-dimer/Albumin ratio in predicting pulmonary embolism.

	Diagnostic Test					ROC Curve		*p*
	Cut-off	Sensitivity	Specifity	PPV	NPV	AUC	(95% CI)	
**D-dimer/** **Albumin**	≥**205.33**	92.1	57.0	54.2	92.9	**0.882** (0.832–0.931)		<0.001 *

AUC: Area under the curve, PPV: Positive predictive value, NPV: Negative predictive value, CI: Confidence interval. * *p* < 0.001 indicates statistical significance.

**Table 3:** Components and scoring system of the Hema-PE Score.

Parameter	Cut-off	Points Assigned
D-dimer/Albumin ratio	>206.5	2
Immobility	≥3 days	1
Central venous catheter	Present	1
C-reactive protein (CRP)	>10 mg/L	1
Platelet count	>350 × 10^9^/L	1
Hemoglobin	<10 g/Dl	1

Risk categories based on total score: -Low risk: 0–2 points, -Intermediate risk: 3–4 points, -High risk: 5–7 points.

**Table 4:** Cut-off values and ROC analysis results of the new scoring system in predicting pulmonary embolism.

	Diagnostic Test					ROC Curve		*p*
	Cut-off	Sensitivity	Specifity	PPV	NPV	AUC	95% CI	
Score 2	≥2	100.0	44.7	50.0	100.0	0.724	0.651–0.796	<0.001 *
Score 3	≥3	**100.0**	**76.3**	**70.0**	**100.0**	**0.882**	**0.832–0.931**	<0.001 *
Score 4	≥4	**57.1**	**95.6**	**87.8**	**80.1**	**0.764**	**0.682–0.845**	<0.001 *
Score 5	≥5	31.7	100.0	100.0	72.6	0.659	0.569–0.749	<0.001 *

Clinically relevant thresholds (≥3 and ≥4) are highlighted in bold. AUC: Area under the curve, PPV: Positive predictive value, NPV: Negative predictive value, CI: Confidence interval. * *p* < 0.001 indicates statistical significance.

## Data Availability

The original contributions presented in this study are included in the article. Further inquiries can be directed to the corresponding author.
